# Bdelloid rotifers (Rotifera, Bdelloidea) of China: diversity and new records

**DOI:** 10.3897/zookeys.941.50465

**Published:** 2020-06-16

**Authors:** Yue Zeng, Nan Wei, Qing Wang, Nataliia S. Iakovenko, Ying Li, Yufeng Yang

**Affiliations:** 1 Institute of Hydrobiology, College of Life Science and Technology, Jinan University, Guangzhou 510632, China Jinan University Guangzhou China; 2 Southern Marine Science and Engineering Guangdong Laboratory (Zhuhai), Zhuhai, 519000, China Southern Marine Science and Engineering Guangdong Laboratory Zhuhai China; 3 South China Institute of Environmental Sciences, Ministry of Ecology and Environment, Guangzhou 510530, China South China Institute of Environmental Sciences Guangzhou China; 4 Czech University of Life Sciences Prague, Faculty of Forestry and Wood Sciences, Kamýcká 129, CZ– 16521 Praha 6– Suchdol, Czech Republic Schmalhausen Institute of Zoology NAS of Ukraine Kyiv Ukraine; 5 Schmalhausen Institute of Zoology NAS of Ukraine, Department of Fauna and Systematics, Bogdana Khmelnyts’kogo 15, 01601 Kyiv, Ukraine Czech University of Life Sciences Prague Prague Czech Republic

**Keywords:** bdelloids, biogeography, morphospecies, Oriental region, taxonomy

## Abstract

Bdelloid rotifers are a group of microscopic invertebrates known for their obligate parthenogenesis and exceptional resistance to extreme environments. Their diversity and distributions are poorly studied in Asia, especially in China. In order to better understand the species distribution and diversity of bdelloid rotifers in China, a scientific surveys of habitats was conducted with 61 samples (both terrestrial and aquatic habitats) from 11 provinces and regions of China, ranging from tropics to subtropics with a specific focus on poorly sampled areas (Oriental) during September 2017 to October 2018. A total of 59 morphospecies (including subspecies) were found, of which, thirty-nine morphospecies (including one genus) are new records for China, almost doubling the number of previous records. Four rare morphospecies (Adineta cf. acuticornis Haigh, *A. beysuna*e Örstan, *Habrotrocha ligula loxoglotta* De Koning and *H. serpens* Donner) are depicted and redescribed, and an updated checklist of Chinese bdelloids with their location and ecological information is presented. This study provides new data from a large region of China, enriching the knowledge of bdelloid biodiversity, and their global biogeography.

## Introduction

Bdelloid rotifers are microscopic invertebrates that constitute a subclass Bdelloidea of the phylum Rotifera, known for their peculiar obligate parthenogenesis (Welch and Meselson 2000; Welch et al. 2004) and outstanding ability to withstand harsh periods through anhydrobiosis (Ricci 1998; Gladyshev and Meselson 2008). The minute size of bdelloids (from less than 160 to 500–600 µm) allows their long-distance dispersal by wind, water, and animals to access to almost all possible habitats (Bohonak and Jenkins 2003; Fenchel 2004; Kellogg and Griffin 2006). They inhabit both aquatic (mainly freshwater lakes, ponds, and streams) and terrestrial habitats (e.g., mosses, lichens, tree barks, soil and litter) (Donner 1965). Rarely, bdelloids are found in marine and brackish waters (Fontaneto et al. 2006; Demirkalp et al. 2010; Song 2014).

Analysis of Bdelloidea taxonomy characteristics is problematic because only observation of living and active specimens allows appropriate identification of species. That is why it has not been widely carried out. Furthermore, there are no readily available reagents that can be used to anesthetize them and preserve their bodies fully extended (Örstan and Plewka 2017). Untill recently, only about 460 bdelloid species have been described worldwidely (Segers 2007), but there is ample evidence that the total number of bdelloid species is at least several times greater than the current one (Fontaneto et al. 2011; Robeson et al. 2011). In addition, the intensity of taxonomic researches on bdelloid species in different regions of the world was extremely uneven, thus the species diversity varies greatly from region to region. For instance, over 300 species are known from Europe (Fontaneto et al. 2007), while only about 50 species are found in the Oriental region (Segers 2007).

In China, only 48 bdelloid morphospecies have been reported (Zhuge et al. 1998; Koste and Zhuge 1998; Yin and Xu 2016) (Table 1). The first study on the Chinese bdelloid rotifers was reported by Thorpe (1893), who found four species of *Rotaria* in Yangtze River area in Wuhu city, Anhui Province. After that, few fragmental reports from fresh waters and terrestrial environments in a large region of China were presented (Daday 1906; Stewart 1908; Gee 1927; Wang 1961; Bartoš 1963; Wang 1974; Gong 1983; Koste and Zhuge 1996, 1998; Zhuge et al. 1998; Yin and Xu 2016). Up to now, this taxon has not been actively studied in China comparing to Europe or even to Antarctica (Segers 2007), and the biogeography of bdelloids in South Asia is unclear, and their habitat preferences are incomplete. This study aimed to conduct a taxonomic work and evaluate the diversity of bdelloid rotifers in China, especially the poorly investigated tropical zones of the Oriental biogeographic region.

**Table 1.** Checklist of bdelloid rotifers recorded from China before 2015.

Species	Habitats	EL (m)	WT (°C)	AT (°C)	pH	Distribution and references
*Adineta gracilis* Janson, 1893	Moss	800–1400	–	–	–	GD (l)
*A. oculata* (Milne, 1886)	Moss	800–1800	–	–	–	GD (l)
*A. vaga* (Davis, 1873)	Moss and stream	0–1750 m	16–18	26–28	5	GD (f, l), TB (g, h)
*Dissotrocha aculeata* (Ehrenberg, 1832)	Pond, river and bog	0–3650	20	20	6	IM (b), HB, SD, ZJ, SC, XJ (e) TB (h), GD (l)
*D. macrostyla* (Ehrenberg, 1832)	Pond and bog	0–3030	17–20	13.5	6	JS (d), TB (h), HA (i)
*D. macrostyla tuberculata* (Gosse, 1886)	Puddle on the roadside	–	20	–	7.6	HA(k)
*Habrotrocha angusticollis angusticollis* (Murray, 1905)	*Sphagnum*, river, lake branch channel and puddle with aquatic plant	0–4750	14–30	21–25.5	6–8.5	ZJ (e), TB (h), HA (i, k), GD (l)
*H. angusticollis attenua*ta (Murray, 1906)	Moss	–	–	–	–	GD (f)
*H. ampulla* (Murray, 1911)	River with macrophyte	–	20	–	6.32	HA (i)
*H. collaris* (Ehrenberg, 1832)	Bog, stream, lake and moss	800–3800	12–19.5	15–25	6–7	TB (h), GD (l)
*H. constricta* (Dujardin, 1841)	–	–	–	–	–	HA (j)
*H. elegans* (Milne, 1886)	Lake	3658	13	15	7	TB (h)
*H. flexicollis Bartoš, 1963	Moss	–	–	–	–	GD (f)
*H. fusca* (Bryce, 1894)	Moss	–	–	–	–	GD (f)
*H. insignis* Bryce, 1915	Moss	–	–	–	–	GD (f)
*H. modesta* Bartoš, 1963	Moss	–	–	–	–	GD (f)
*H. munda* Bryce,1913	Bog	4200	16.5	13.5	6	TB (h)
*H. perforata* (Murray, 1906)	Moss	–	–	–	–	GD (f)
*H. pulchra* (Murray, 1905)	Spring with attachment from meadow, stone and soil, puddle from glacier	5700	17	11	8	TB (g, h)
*H. pusilla* (Bryce, 1893)	Puddle from spring and wet moss on stone	830–2400	30	25	6	TB (h)
*H. thienemanni* Hauer, 1924	Puddle with aquatic plant and moss, glacier	830–5550	13–30	15–25	5–7	TB (g, h)
*H. tridens* (Milne, 1886)	Moss	600–1900	–	–	–	GD (l)
Otostephanos cf. donneri (Bartoš, 1959)	Aquatic ecosystem	–	–	–	–	YN (j)
*Macrotrachela bullata* (Murray, 1906)	Stream with algae or moss	1668–4150	10–18	19–28	5–6	TB (g, h)
*M. ehrenbergii* (Janson, 1893)	Moss	4500	–	–	–	TB (h)
*M. insolita* De Koning, 1947	Moss	1000–1200	–	–	–	GD (l)
*M. multispinosa* Thompson, 1892	Attachments on aquatic plants, bogs and moss from grass lands	3300	16	17	6	TB (h)
*M. musculosa* (Miline, 1886)	Springs and wet moss	4150–4500	6	11–19	6	TB (h)
*M. plicata* (Bryce, 1892)	Puddles	4400–4500	12–14	10–14	7	TB (h)
*M. papillosa* Thompson, 1892	Moss	–	–	–	–	GD (f)
*M. punctata* (Murray, 1911)	Attachment from stone and wet grass	3800–3850	12	19	7	TB (h)
*M. quadrlcornlfera* Milne, 1886	Moss	0–1900	–	–	–	GD (l)
*Mniobia tentans* Donner, 1949	Stream with algae or moss	1668–1750	16–18	25–28	5	TB (g, h)
*Philodina citrina* Ehrenberg, 1832	Rice field, puddle, shallow and wet moss	600–4350	12–27	10–28	6–7	TB(c, h), GD (l)
*P. erythrophthalma* Ehrenberg, 1830	Pond, pool and stream with algae	0–3370	9	12	7	HB (e), TB (c, h)
*P. megalotrocha* Ehrenberg, 1832	Lake with macrophyte, pond, water reservoir and rice field	–	20–26	–	6–8	HB, SH, JS, ZJ (e) HA(i, k)
*P. nemoralis* Bryce, 1903	Rice field, bog and moss	2000–2400	36	29	5	TB (h)
*P. roseola* Ehrenberg, 1832	River, pond, marsh and moss	0–3100	–	–	–	IM (b) TB(c), HB, SH, JS, ZJ, HA (e) GD (l)
*P. vorax* (Janson, 1893)	Stream, spring and puddle from river or glacier	2400–5500	7–17	–	6–8	TB (g, h)
*Pleuretra similis Bartoš, 1963	Moss	–	–	–	–	GD (f)
*Rotaria citrina* (Ehrenberg, 1838)	Rice field and pool	0–2400	13–28	–	6	HB (e), TB (h)
*R. macroceros* (Gosse, 1851)	Yangtze River, lake and moss	–	25	–	6	AH (a), HB (e), GD (l), HA(i, k)
*R. macrura* (Ehrenberg, 1832)	River	–	–	–	–	IM (b)
*R. neptunia* (Ehrenberg, 1830)	Pond, rice field and puddle	0–3650	18–26	–	6–8.7	AH (a), SH, JS, ZJ, HB, BJ, HL, LN, GS, HN, GD, GX, YN, SC (e), HA (i, k), TB (h)
*R. rotatoria* (Pallas, 1766)	Pond and rice field	0–830	20–26	–	6–8.7	AH (a), IM (b), SH, HB (e), TB (h), HA (i, k)
*R. sordida* (Western, 1893)	Moss; polluted lake	–	21	–	7.1	GD (f), HA(i, k)
*R. tardigrada* (Ehrenberg,1830)	Lake, polluted river and puddle	0–3658	18–25	–	6–7	AH (a), HA (i, k), HL, SH, GS, JS (e) TB (h)
*R. tridens* (Montet, 1915)	Bog, wet moss pool and attachment from stone	2900–4550	–	15–18	6	TB (c, h)

Sources: (a) (Thorpe 1893); (b) (Daday 1906); (c) (Stewart 1908); (d) (Gee 1927); (e) (Wang 1961); (f) (Bartoš 1963); (g) (Wang 1974); (h) (Gong 1983); (i) (Koste and Zhuge 1996); (j) (Zhuge 1997); (k) (Koste and Zhuge 1998); (l) (Yin and Xu 2016). ‘cf.’ is retained for those taxa which have some differences from the nominate morphospecies, requiring further study. *: China only. Abbreviation: AH: Anhui; AT: air temperature; BJ: Beijing; EL: elevation; GD: Guangdong; GS: Gansu; GX: Guangxi; HA: Hainan; HB: Hubei; HL: Heilongjiang; HN: Hunan; IN: Inner Mongolia; JS: Jiangsu; LN: Liaoning; SD: Shandong; SC: Sichuan; SH: Shanghai; TB: Tibet; WT: water temperature; XJ: Xinjiang; YN: Yunnan; ZJ: Zhejiang.

## Materials and methods

### Sampling area, collection procedures and sample processing

**Figure 1.**
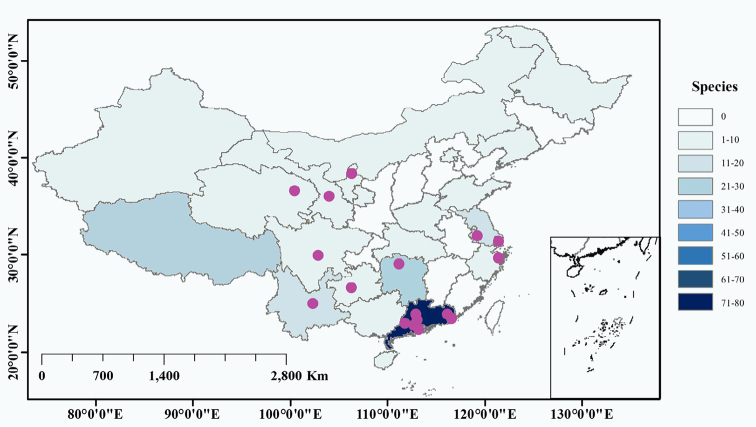
Locations of the sampling sites in this study (purple circles) and species richness of bdelloid rotifers (blue) recorded between 1908 and 2018 in China.

A total of 61 samples was collected during the period from September 2017 to October 2018 in 11 provinces and regions of China across its subtropical and tropical zones at altitudes from 0–2850 m above sea level from four types of terrestrial habitat (soil, mosses, leaf litter and lichens) and four types of aquatic habitat (plankton, benthos, periphyton and dew) in fresh or brackish waters (Fig. 1, Table 2). Of these samples, eleven were collected from fresh water, six from brackish water, one from dew on leaves, thirty from mosses, ten from leaf litter, two from lichens, and one from soil with mosses.

**Table 2.** Sampling locality information of this survey.

Locality codes	Locality	Sampling date	Habitat	GPS coordinates	Elevation (m)
GD1	Chaozhou	18.08.2017	Moss on concrete	23°58'15.13"N, 116°38'12.14"E	1136
GD2	Chaozhou	18.08.2017	Moss on bark	23°58'14.93"N, 116°38'12.08E	1139
GD3	Chaozhou	18.08.2017	Moss on rock	23°58'14.99"N, 116°38'12.11"E	1138
GD4	Chaozhou	18.08.2017	Moss on soil	23°58'15.02"N, 116°38'12.09"E	1138
GD5	Chaozhou	18.08.2017	Moss on rock	23°55'59.37"N, 116°36'59.84"E	436
GD6	Guangzhou	05.11.2017	Dry moss on bark	23°06'35.11"N, 113°14'21.20"E	10
GD7	Guangzhou	20.09.2017	Lotus pond	23°07'54.80"N, 113°20'39.44"E	16
GD8	Guangzhou	05.11.2017	Lotus pond	23°07'54.80"N, 113°20'39.44"E	16
GD9	Guangzhou	25.10.2018	Lotus pond	23°07'54.80"N, 113°20'39.44"E	16
GD10	Guangzhou	11.06.2018	Moss on concrete	23°08'1.29"N, 113°20'38.81"E	15
GD11	Guangzhou	11.06.2018	Soil	23°07'51.89"N, 113°20'37.45"E	18
GD12	Haiou island	28.10.2017	Water hyacinth root in brackish water	22°58'23.36"N, 113°30'40.95"E	4
GD13	Guangzhou	13.06.2018	Bamboo leaf litter	23°18'4.12"N, 113°26'23.21"E	214
GD14	Guangzhou	13.06.2018	Bamboo leaf litter	23°18'18.99"N, 113°26'56.14"E	152
GD15	Guangzhou	20.06.2017	Bottom of lotic water	23°18'0.59"N, 113°26'27.47"E	226
GD16	Guangzhou	26.10.2018	Urban river	23°03'29.0"N, 113°24' 26.6"E	0.75
GD17	Nanao island	22.04.2018	Puddle	23°25'44.88"N, 117°01'49.56"E	108
GD18	Nanao island	09.01.2018	*Gracilaria lichenoides* in brackish pond	23°27‘18.13“N, 117°7'31.35"E	170
GD19	Nanao island	22.04.2018	Lotic water	23°26'38.29"N, 117°05'22.94"E	124
GD20	Qingyuan	12.05.2018	Moss on concrete	24°36'46.73"N, 112°35'57.02"E	237
GD21	Qingyuan	12.05.2018	Moss on concrete	24°36'40.72"N, 112°35'50.11"E	143
GD22	Qingyuan	12.05.2018	Moss on soil	24°36'40.44"N, 112°36'9.26"E	142
GD23	Qingyuan	12.05.2018	Moss on bark	24°36'41.29"N, 112°36'9.26"E	175
GD24	Qiao island	29.10.2017	Bottom of brackish pool in mangrove	23°27'32.41"N, 117°06'3.59"E	53
GD25	Nanao island	18.11.2018	Leaf litter	22°25'42.45"N, 113°37'51.53"E	137
GD26	Nanao island	18.11.2018	Leaf litter	23°27‘18.13“N, 117°7'31.35"E	9
GS1	Lanzhou	07.06.2018	Wet moss near pond	36°08'25.56"N, 103°41'41.18"E	1615
GZ1	Guiyang	24.08.2017	Moss on rock	26°36'1.75"N, 106°41'10.39"E	1213
GZ2	Guiyang	24.08.2017	Moss on rock	26°05'52.74"N, 105°52'55.89"E	1170
HN1	Changde	20.06.2017	Moss on rock	29°3'10.0"N, 111°40'13"E	31
HN2	Changde	15.09.2017	Moss on rock	29°3'10.0"N, 111°40'13"E	31
HN3	Changde	11.12.2017	Moss on rock	29°3'10.0"N, 111°40'13"E	31
HN4	Changde	12.03.2018	Moss on rock	29°3'10.0"N, 111°40'13"E	31
HN5	Changde	11.12.2017	Aquatic plant	29°02'23.49"N, 111°42'33.35"E	35
HN6	Changde	12.12.2017	*Lemna minor* in river	29°7'20.0"N, 111°39'49"E	57
HN7	Changde	15.09.2017	Water sample from a pond	29°3'10"N, 111°40'13.0"E	30
HN8	Changde	12.03.2018	Moss on soil	29°03'13.78"N, 111°40'12.69"E	31
HN9	Changde	11.12.2017	Lotus pond	29°03'3.68"N, 111°39'57.93"E	35
JS1	Nanjing	15.08.2018	Bamboo leaf litter	32°3'28.63"N, 118°45'27.47"E	39
JS2	Nanjing	15.08.2018	Moss with leaf litter	32°3'28.19"N, 118°45'24.52"E	34
NX1	Yinchuan	02.07.2018	Moss from dessert (32 °C of soil surface)	38°33'45.38"N, 106°32'0.37"E	1128
NX2	Yinchuan	01.07.2018	Extremely dry *Juniperus* litter	38°29'22"N, 106°12'1"E	1109
QH1	Qinghai lake	09.06.2018	Wet moss on bark	36°47'46.11"N, 101°06'18.99"E	2850
SC1	Wawu mountain	23.08.2017	Wet moss on bark	29°40'15.26"N, 102°56'53.92"E	2105
SC2	Wawu mountain	23.08.2017	Wet moss on bark	29°40'10.36"N, 102°56'53.92"E	2105
SC3	Wawu mountain	23.08.2017	Wet moss on bark	29°40'10.36"N, 102°56'53.92"E	2100
SH1	Chongming island	29.12.2017	Aquatic plants in brackish water	31°31'9.5"N, 121°56'4.3"E	3
SH2	Chongming island	29.12.2017	Moss on soil in brackish marsh	31°29'54"N, 121°55'20.7"E	3
SH3	Chongming island	29.12.2017	Reed root in brackish water	31°30'43.9"N, 121°57'27.9"E	3
SH4	Chongming island	29.12.2017	Aquatic plants in brackish water	31°31'2.9"N, 121°55'3.1"E	2
YN1	Kunming	01.06.2018	Moss on concrete	25°3'20.5"N, 102°42'8.6"E	1889
YN2	Kunming	01.06.2018	Moss on soil	25°3‘2.2"N, 102°42'5.1"E	1908
YN3	Kunming	01.06.2018	Moss on concrete	25°3‘6.1"N, 102°42'5.41"E	1900
YN4	Kunming	01.06.2018	Lichens on bark	25°8'0.2"N, 102°39'40.6"E	1900
YN5	Kunming	01.06.2018	Moss on rock	24°57'59.4"N, 102°39'35"E	1888
YN6	Kunming	11.10.2018	Lichens on rock	24°57'49.1"N, 102°37'44.6"E	2150
YN7	Kunming	11.10.2018	Leaf litter	24°57'53.2"N, 102°37'44.6"E	2143
YN8	Kunming	11.10.2018	Dew on leaves	24°57'55.5"N, 102°37'44.3"E	2136
ZJ1	Hangzhou	19.11.2017	Dry moss on *Torreya grandis*’ bark	30°21'42.0"N, 119°34'28"E	305
ZJ2	Ningbo	03.11.2018	Bamboo leaf litter,	29°52'40.3"N, 121°33'15.55"E	37
ZJ3	Zhoushan	03.11.2018	Leaf litter	–	–

GPS coordinates based on WGS84 system. Abbreviation: GD: Guangdong; GS: Gansu; GZ: Guizhou; HN: Hunan; JS: Jiangsu; NX: Ningxia; QH: Qinghai; SC: Sichuan; SH: Shanghai; XJ: Xinjiang; YN: Yunnan; ZJ: Zhejiang.

Based on the definition of boundary between the Palearctic and Oriental biogeographic regions in China (Norton et al. 2010), fifty-seven samples were collected in the Oriental region, while four samples (NX1, NX2, GS1, QH1) were collected in the Palearctic region (Table 2). According to the Geodetector model to partition subtropical and tropical zone in China (Dong 2017), forty-two samples were collected in the subtropical zone, while nineteen samples (GD1–19) were collected in the tropical zone (Table 2).

Samples from terrestrial habitats were placed into firmly closed paper envelopes, then dried at room temperature and stored in the envelopes for several weeks or months. Planktonic samples were obtained by filtering 1 to 5 liters of water through a plankton net with a mesh size 30 µm. Benthic ones were collected by scraping the bottom of water bodies with a 500 ml plastic bottle. Periphytic rotifers were obtained by shaking or scraping aquatic plants, then preserved in plastic bottles.

Samples from aquatic habitats were concentrated by a nylon net of 30 μm mesh size, then examined in lab immediately without fixating or anesthetization. Rotifers from mosses, lichens and leaf litter were extracted by washing the substrate with distilled water following the method of Peters (1993). Soil rotifers were extracted by the method of wet-sieving and centrifugation in a sugar gradient (Freckman 1993).

### Light microscopy procedures

Rotifers isolated from waters were transferred into a Petri dish and sorted under a dark field dissecting microscope (SZX10, Olympus, Japan) with a magnification of 64×. Selected specimens were placed onto glass slides by using micropipettes, then examined alive under a microscope (BX51, Olympus, Japan) with magnification of ×200–400. All living specimens were recorded and photographed using a digital camera (Truechrome Metrics, China) with the software of TCapture. Photos and digital screenshots from videos were used for species identification and illustrations.

### Species identification

Species were identified by both external morphology and anatomy using the keys of Donner (1965) and the original descriptions and redescriptions of specific species (Murray 1906; Song and Kim 2000; Yakovenko 2000a, 2000b; Kutikova 2005; Bielańska-Grajner 2013; Song 2014, 2015; Song and Min 2015; Song and Lee 2017). Drawings of some rare morphospecies were made with Adobe Illustrator CC 2018 and Photoshop CC 2017.

All rotifers were measured from screenshots of digital videos after Iakovenko et al. (2013, 2015) and Örstan (2018). Total length (TL) in the case of adinetid rotifers is the distance between the middle of the anterior rim of the head excluding rostrum, and the posterior rim of the spur pseudosegment; head length (HL) is the distance between the anterior edge of the head (posterior to the rostrum) and the anterior rim of the antennal pseudosegment, i.e., TL and HL do not include the rostrum, because it was usually bent under the head (Iakovenko et al. 2015). The head length in *A. beysunae* is the distance between the anterior edge of the head and an imaginary line passing through the innermost denticles of the rakes to better compare it with the original description (Örstan 2018). The number of denticles on each rake is represented formulaically using an ‘en dash’ (Örstan 2018). We counted the distal foot with the toes as a pseudosegment separate from the one carrying the spurs as Bryce (1894), Donner (1965) and Iakovenko et al. (2013, 2015) did.

### Abbreviations

BW body width (when creeping)

CW corona width

FL foot length

FW foot width

HL head length

HW head width

MinNW minimal neck width

MxNW maximal neck width

NL neck length

TL total length

TrL trunk and rump length

RaL ramus length

RkW rake width

RL rump length

RW rump width

SL spur length

SSW spur pseudosegment width

TrW trophi width

## Results

### Species diversity

Fifty-nine morphospecies (including three subspecies) were identified in this survey (Table 3), and the bdelloids that were unidentifiable to the species level were not included in the list. Of them, thirty-nine taxa (including one genus) are new records for China, and thirty-eight species are new records for the Oriental region. The species list of Chinese bdelloid fauna has been increased from 48 to 87. Detailed information about their distribution and ecological information is reported in Tables 2, 3.

**Table 3.** Bdelloid rotifers found in this study with their updated biogeographic distribution after Segers (2007).

Species	Locality codes	Biogeographic regions
^*^Adineta cf. acuticornis Haigh, 1967	GD6, YN5–6, SC2	AUS, ORI^#^
^*^A. barbata Janson, 1893	GD10, 14, JS1, ZJ2	AFR, ANT, AUS, NEA, NEO, PAL, ORI^#^
^*^A. bartosi Wulfert, 1960	GZ2	PAL, ORI^#^
^*^A. beysunae Örstan, 2018	GD13–14, 25–26, YN7–8, JS1	NEA, ORI^#^
^*^A. cuneata Milne, 1916	GD1–2, SC2, JS2, YN6–7	AFR, AUS, NEA, PAL, ORI^#^
*A. gracilis* Janson, 1893	HN2, QH1, JS1	AFR, ANT, AUS, NEA, ORI, PAL
*A. oculata* (Milne, 1886)	GD7, YN3, HN1	NEO, PAL, ORI^#^
^*^A. ricciae Segers & Shiel, 2005	GD23, HN4	AUS, ORI^#^
^*^A. steineri Bartoš, 1951	GD13	ANT, AUS, NEA, NEO, PAL, ORI^#^
*A. vaga* (Davis, 1873)	GZ2, HN2–4, ZJ2, YN1,7, GD5,7,13–14, 20–21,23	AFR, ANT, AUS, NEA, NEO, ORI, PAL
*Dissotrocha macrostyla* (Ehrenberg, 1838)	HN6	AFR, AUS, NEA, NEO, ORI, PAL
^*^Habrotrocha bidens (Gosse, 1851)	ZJ1	AFR, AUS, NEA, NEO, ORI, PAL
^*^H. cf. spicula Bryce, 1913	GD2	AFR, AUS, ORI, PAL
*H. constricta* (Dujardin, 1841)	HN2	AFR, ANT, AUS, NEA, NEO, PAC, PAL, ORI^#^
*H. insignis* Bryce, 1915	GD3	AUS, PAL, ORI^#^
^*^H. ligula loxoglotta De Koning, 1947	YN5	PAL, ORI^#^
^*^H. rosa Donner, 1949	GD25	AFR, AUS, NEA, NEO, PAL, ORI^#^
^*^H. serpens Donner, 1949	GD6	AFR, AUS, PAL, ORI^#^
^*^Otostephanos regalis Milne, 1916	GD13	AFR, PAL, ORI^#^
^*^Scepanotrocha semitecta Donner, 1951	SC1	NEO, PAL, ORI^#^
*Macrotrachela bullata* (Murray, 1906)	GD3–4, GZ2	AFR, ORI, PAL
*M. ehrenbergii* (Janson, 1893)	HN7, GZ1	AFR, AUS, NEA, NEO, ORI, PAC, PAL
^*^M. habita (Bryce, 1894)	GD6, 11,20, 22–23, YN1–3, GZ1	AFR, ANT, AUS, NEA, NEO, ORI, PAL
^*^M. hewitti (Murray, 1911)	SH1	AFR, PAL, ORI^#^
^*^M. inermis Donner, 1965	YN4	PAL, ORI^#^
*M. insolita* De Koning, 1947	GD2, HN8	ANT, AUS, NEA, NEO, PAL, ORI^#^
^*^M. latior Doner, 1951	YN7	PAL, ORI^#^
^*^M. libera Donner, 1949	HN4	PAL, ORI^#^
*M. multispinosa multispinosa* Thompson, 1892	GD6	AFR, AUS, NEA, NEO, ORI, PAL
^*^M. multispinosa brevispinosa (Murray, 1908)	YN5	AFR, AUS, NEO, ORI, PAL
^*^M. nana (Bryce, 1912)	QH1	AFR, AUS, NEA, NEO, PAL
*M. plicata* (Bryce, 1892)	SC2–3	AFR, AUS, NEA, PAL, ORI^#^
^*^M. quadricornifera quadricorniferoides De Koning, 1929	JS1, 2	AFR, ANT, NEO, ORI, PAL
^*^M. quadricornifera scutellata Schulte, 1954	GD13	AUS, PAL, ORI^#^
^*^M. timida Milne, 1916	SC1–3, YN7	AFR, AUS, PAL, ORI^#^
^*^Philodina acuticornis Murray, 1902	GD20–21, JS1, ZJ2	AFR, AUS, NEA, NEO, PAL, ORI^#^
^*^P. cf. indica Murray, 1906	YN4	NEA, PAL, ORI^#^
^*^P. cf. proterva Milne, 1916	GD5, YN1, 6, ZJ2	AFR, AUS, NEA, PAL, ORI^#^
^*^P. childi Milne, 1916	GD14, YN7	PAL, ORI^#^
^*^P. duplicalcar (De Koning, 1947)	NX2	PAL
*P. megalotrocha* Ehrenberg, 1832	HN5–6, 9, GD9, 12	AFR, AUS, NEA, NEO, ORI, PAL
^*^P. cf. parvicalcar De Koning, 1947	SH2, GD25	PAL, ORI^#^
^*^P. plena (Bryce, 1894)	QH1, YN7	AFR, ANT, AUS, NEA, NEO, PAL, ORI^#^
^*^P. rapida Milne, 1916	YN7	AFR, NEO, PAL, ORI^#^
*P. roseola* Ehrenberg, 1832	GD19	AFR, AUS, NEA, NEO, PAL, ORI^#^
^*^P. rugosa Bryce, 1903	GD20–21	AFR, AUS, NEA, NEO, PAL, ORI^#^
^*^P. tenuicalcar De Koning, 1947	NX1	PAL
^*^P. tranquilla Wulfert, 1942	HN2, GS1	AUS, PAL, ORI^#^
*P. vorax* (Janson, 1893)	HN2	AFR, AUS, NEA, NEO, ORI, PAL
^*^Pleuretra africana Murray, 1911	YN2, 6	AFR, NEO, ORI^#^
^*^P. brycei (Weber, 1898)	GD15, 23	AFR, AUS, NEA, NEO, PAL, ORI^#^
*Rotaria citrina* (Ehrenberg, 1838)	GD16	AFR, AUS, NEA, PAL, ORI^#^
^*^R. laticeps Wulfert, 1942	GD15, 24	AUS, PAL, ORI^#^
*R. neptunia* Ehrenberg, 1830	GD16–17	AFR, AUS, NEA, NEO, ORI, PAL
^*^R. neptunoida Harring, 1913	GD16–17, 19	AFR, AUS, NEA, ORI, PAL
*R. rotatoria* (Pallas, 1766)	HN5, GD8, 18, SH1, 3	AFR, AUS, NEA, NEO, ORI, PAL
*R. sordida* (Western, 1893)	HN2, 8, YN2–3, GD13–14,26, JS1	AFR, AUS, NEA, NEO, ORI, PAL
*R. tardigrada* (Ehrenberg, 1830)	HN9	AFR, AUS, NEA, NEO, ORI, PAL
*R. tridens* (Montet, 1915)	HN6, 9, GD9, 12	AUS, NEA, NEO, PA^L^, ORI#

*: Taxa new for China, #: new for ORI. Abbreviations: AFR: Afrotropical region; ANT: Antarctic region; AUS: Australian region; NEA: Nearctic region; NEO: Neotropical region; ORI: Oriental region; PAC: Pacific region; PAL: Palearctic region. ‘cf.’ is retained for those taxa which have some differences from the nominate morphospecies, requiring further study. Locality codes see Table 2 for sampling information.

During our survey, five collected bdelloids have a general resemblance to known species, but also showed some dissimilar traits from previously described taxa, and they were qualified with ‘cf.’ and await further analysis. One of these doubtful species, reported as H. cf. spicula Bryce, which showed a upturned dorsal protrusion. Philodina cf. indica Murray, P. cf. proterva Milne and P. cf. parvicalcar showed relative wide range of variations in their head proportion, which need further analyses.

Among these new records, some species are very rare, and few were first found out of their type localities or habitats, e.g., *Adineta beysunae* Örstan and *Habrotrocha ligula loxoglotta* De Koning; some new morphological characteristics were observed and need to be added to the original descriptions, e.g., Adineta cf. acuticornis Haigh and *Habrotrocha serpens* Donner, which are redescribed and illustrated in the next section.

Species richness of bdelliods recorded between 1908 and 2018 in different provinces of China is presented in Figure 1, showing that sampling intensity greatly influenced the species diversity in different regions of China. For instance, the provinces of Guangdong, Yunnan and Hunan were the subject of 26, eight, and nine studies, which recorded 33, 18, and 16 morphospecies, respectively, whereas the provinces of Jiangsu, Zhejiang, Guizhou, Sichuan, Shanghai, Qinghai, Ningxia, and Gansu have no more than four investigations, which only recorded up to six morphospecies for each.

### Redescriptions of some rare morphospecies

**Phylum Rotifera Cuvier, 1817**

**Class Eurotatoria De Ridder, 1957**

**Order Adinetida Melone & Ricci, 1995**

**Family Adinetidae Melone & Ricci, 1995**

**Genus *Adineta* Hudson & Gosse, 1886**

#### Adineta cf. acuticornis

Taxon classificationAnimaliaBdelloideaAdinetidae

Haigh, 1967

BBD9A6D0-B51A-56BD-9CCF-4D393202A1C0

Figure 2 ; Table 3

##### Material.

Eight specimens found in mosses and two specimens found in lichens, from tropical (GD 6) and subtropical (YN 5–6, SC 2) zones (Table 2).

##### Description.

Body transparent and colorless, with smooth skin. No eyespots. Rostrum rather long when animal creeps and stretches out, distal rostral pseudosegment semi-circular and flattened. Rostral lamella divided into two broad sickles-like lobes, immobile, laterally elongated, no trace of cilia under the present microscope image. Small oval head, HW 63–90% of HL and 11–16% of TL, HL 15–18% of TL. Five rectangular denticles in each rake.

**Figure 2.**
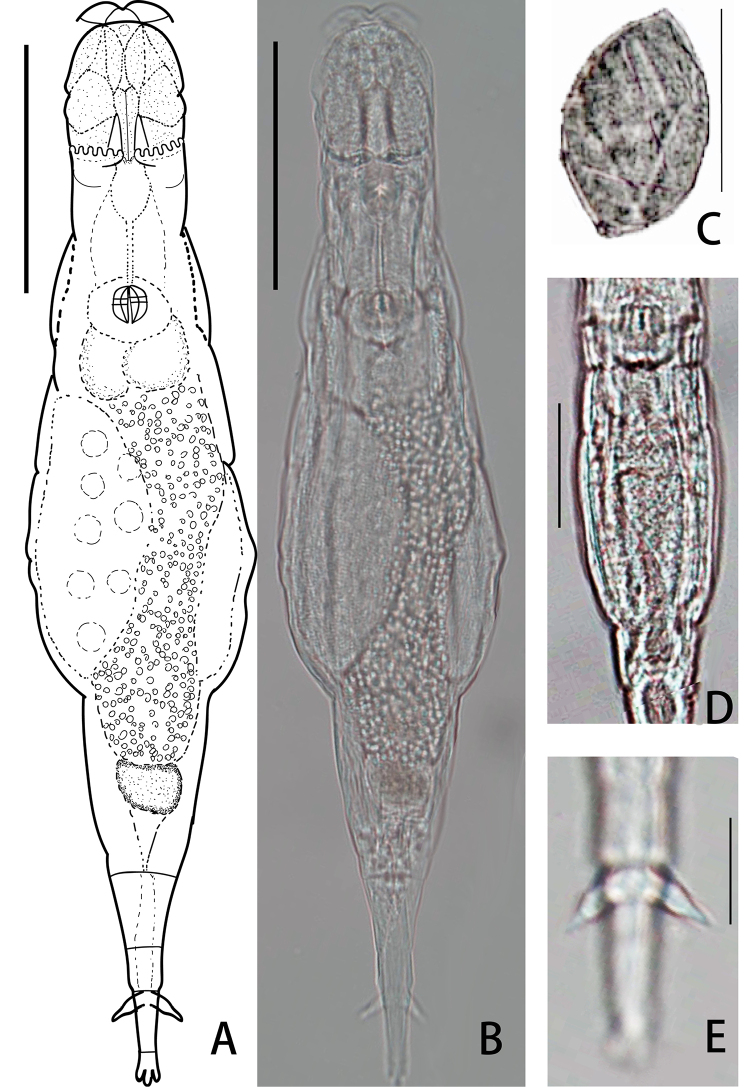
Adineta cf. acuticornis Haigh, 1967 **A, B** habitus, ventral view **C** egg **D** stomach lumen **E** spur. Scale bars: 50 μm (**A–D**); 10 μm (**E**).

Neck width not distinct from head and trunk. The width of the first two pseudosegments of neck approximately equal to HW, the second neck pseudosegment much wider and swollen than the first one. Antenna of two pseudosegments, with length 56–64% of the bearing pseudosegment width. Trunk oval, BW 15–22% of TL. Rump conical, TrL 54–67% of TL. The stomach lumen very narrow and Z-shaped (Fig. 2D). Oviparous; egg oval and smooth, one knob at each pole (Fig. 2C); Vitellarium large with eight nuclei.

Foot slim and short, of four pseudosegments. Spurs long, the inner edge of the spurs almost parallel to the straight outer edge for two-thirds of its length, then a small bulge followed by a contraction and tapers to a sharp point (Fig. 2E). SL 4–8% of TL, and 143–193% of SSW. Three long and unsegmented toes. Dorsal toe longer than two ventral toes. Trophi small, round. Dental formula 2/2.

##### Measurements.

The detailed measurements are summarized in Table 4 with a comparison of the original data from Haigh (1967).

**Table 4.** Comparison the body dimensions of Adineta acuticornis between Chinese specimens and the original description.

**Measurements**	**Chinese specimens**	**Original description**
TL	166–266 (227±33)	210
BW	30–61 (44±10)	
HL	30–45 (40±5)	
HW	25–37 (30±4)	30
NL	15–34 (25±6)	
MinNW	16–31 (25±4)	
MxNW	22–42 (31±6)	
RL	20–44 (30±9)	
RW	22–38 (25±7)	
FL	21–32 (28±4)	
FW	11–16 (13±2)	
SL	11–13 (12±1)	12
SSW	6–8 (7±1)	9
RaL	9–12 (10±1)	
TrW	5–6 (5±1)	7.5
Rake	5–5	
TL/SL	14.4–21.6 (18.3±3.4)	17.5
TL/HW	6.6–8.6 (7.5±0.8)	7
Rostral lamella	immobile	immobile
Antenna	1/2 MNW	half neck width
Foot segments	4	4
Stomach lumen	one loop	two loops
Habitats	lichen and moss	damp moss on soil

BW: body width; FL: foot length; FW: foot width; HL: head length; HW: head width; MinNW: minimal neck width; MxNW: maximal neck width; NL: neck length; RaL: ramus length; RL: rump length; RW: rump width; SL: spur length; SSW: spur pseudosegment width; TL: total length; TrW: trophi width. Measurements are given in μm.

##### Remarks.

*Adineta acuticornis* has not been found since its original description by Haigh (1967) and was considered as an endemic morphospecies of New Zealand (Shiel and Green 1996). It was found in China for the firstly time also in the Oriental biogeographic region recorded in two provinces of China in 2017 or 2018. It was recorded in damp mosses on soil face in the type locality, whereas in this study, numerous specimens were recorded in both dry and damp mosses, and two specimens in lichens on soil surface.

A distinct characteristic differentiating this morphospecies from *Adineta vaga* Davis is its wide and rostral lamellae which are slightly wider than the anterior head, while the rostral lamellae of *A. vaga* are narrower than the anterior head. It differs from *Adineta glauca* Wulfert by its spur shape, which is short and has a flat base, while *A. glauca* spur with a swollen base. This morphospecies differs from *Adineta longicornis* Murray by its spur shape which has bulge, while *A. longicornis* spur is slender and acute (Murray 1906: 5a, 5b).

The general morphology of the Chinese specimen conforms to the description of the New Zealand population, except the position of the spur contraction is closer to the tip (the contraction is in the middle of the spur in Haigh’s description) and the stomach lumen do not have distinct two loops as Haigh’s description. A comparison with Haigh’s (1967) body dimensions showed a similar body proportion (Table 4). Since there was no genetic evidence to prove it actual systematic status, we assigned ‘cf.’ (resembling original description) as the status of this find. Besides, we observed three new morphological features missed by Haigh (1967): each rake with five denticles, a larger vitellarium with eight nuclei and egg with one knob on each pole.

#### Adineta beysunae

Taxon classificationAnimaliaBdelloideaAdinetidae

Örstan, 2018

DB0A782F-7F55-5C75-8502-6CD9AB3A05E8

Figure 3 ; Table 3

##### Material.

Numerous specimens found in leaf litter from three provinces (GD13–14, 25–26, YN7, JS1) across tropical and subtropical zones. One specimen found in dew on leaves from Southwest of China (YN8) (Table 2).

##### Description.

Body angulate, large and transparent. Sometimes the organs in the trunk show brown coloration. No eyespots. Rostral lamella flat and widened, with two lateral triangular auricular protrusions holding long rostral setae under them (the number of stiff under each could not be counted under microscope). Setae length varies from 11 to 30 μm. Head trapezoid, rather large and long, HW 80–110% of HL^b^, HL^b^ 17–22% of TL, HW 13–20% of TL. Numbers of U-gaps denticles on rakes: 9–9 (*N* = 3), 10–10 (*N* = 4).

**Figure 3.**
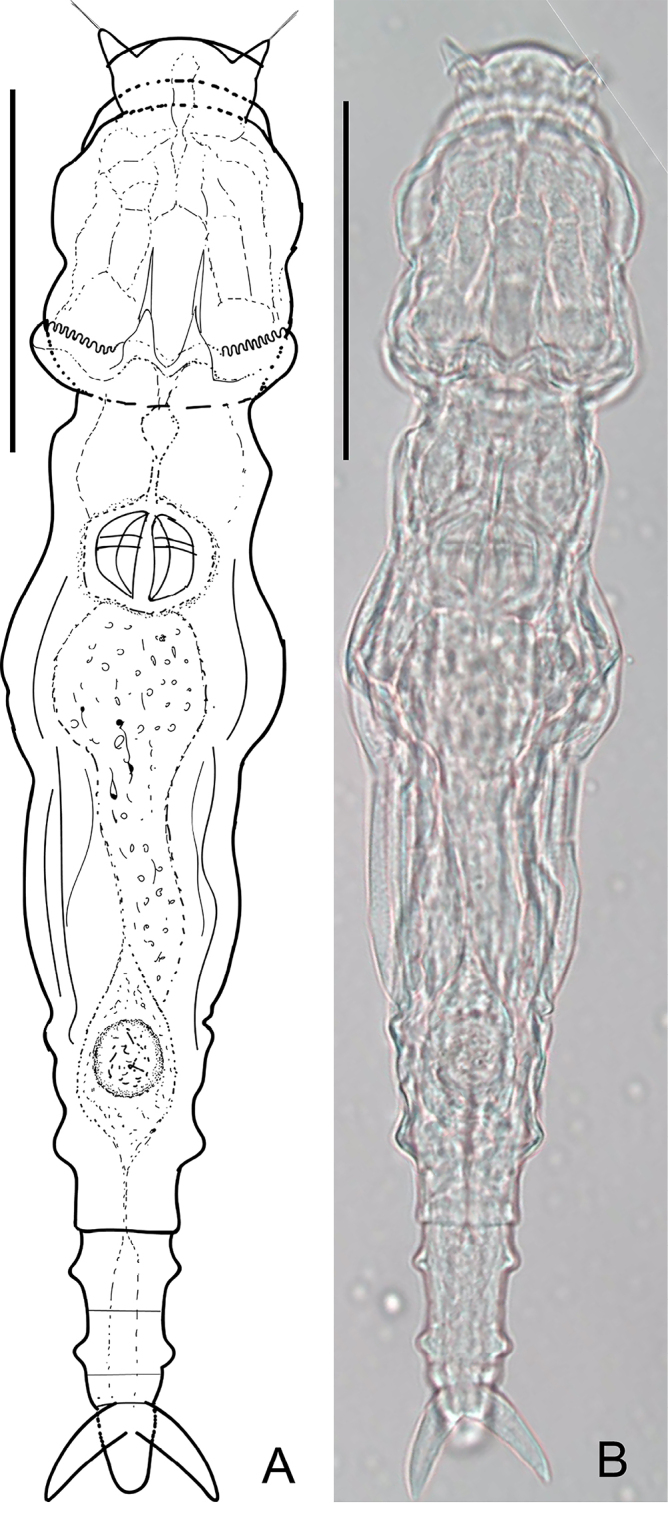
*Adineta beysunae* Örstan, 2018 **A, B** habitus, dorsal view. Scale bars: 50 μm (**A, B**).

Neck distinct from head, the first two pseudosegments of neck narrower than HW. Trunk oval. Posterior end of the first rump pseudosegment with a pair of lateral angular knobs.

Foot of five pseudosegments with two pairs of lateral knobs on its first two pseudosegments, FL 14–22% of TL. Spurs long and sturdy, with short interspace, SL 6–8% of TL, 172–284% of SSW. Three short unsegmented toes. Ventral toe longer than two dorsal toes. Dental formula 2/2.

##### Measurements.

TL 289±40 μm, HL^b^ 49±5 μm, HW 45±4 μm, FL 49±8 μm, SL 20±1 μm, SSW 10±1 μm, RkW (*N* = 2, with 9–9 denticles; *N* = 4, with 10–10 denticles) 21±1 μm, RaL (*N* = 14) 15.9±2 μm, TrW 7.3±1 μm.

##### Remarks.

This is the second report of this morphospecies since its original description by Örstan (2018) in rainwater and plant debris from the United States. In the present study, *A. beysunae* was found in leaf litter and dew on leaves. And interestingly, it was abundant in 60% of all leaf litter samples. Our study suggested *A. beysunae* might have a habitat preference for leaf litter and temporary waterbodies.

**Family Habrotrochidae Harring, 1913**

**Genus *Habrotrocha* Ehrenberg, 1838**

#### Habrotrocha ligula loxoglotta

Taxon classificationAnimaliaBdelloideaHabrotrochidae

De Koning, 1947

403B6D29-02A8-512B-B905-F01C9E43B440

Figure 4 ; Table 3

##### Materials.

Five specimens found in mosses on rock from Southwest China (YN5) (Table 2).

##### Description.

**Figure 4.**
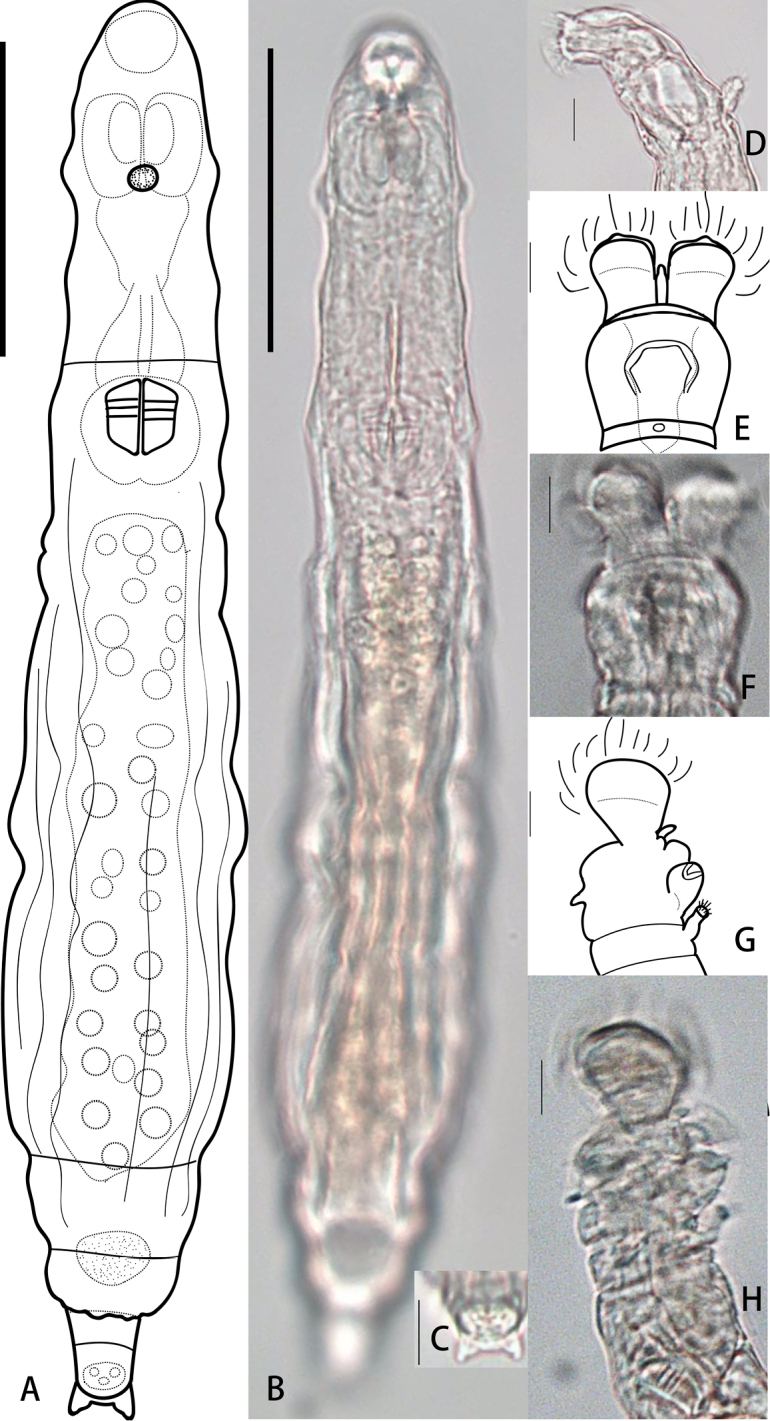
*Habrotrocha ligula loxoglotta* De Koning 1947 **A, B** habitus, creeping, dorsal view **C** rostrum, lateral view **D, E** head, dorsal view **F, G** head, with ligula sloping obliquely to the dorsal side, lateral view. Scale bars: 50 μm (**A, B**), 10 μm (**C–G**).

Body slender and transparent, integument smooth. Rostrum long and strongly bent ventrally. Rostral lamellae divided into two semi-circular lobes and wider than the anterior rim of rostrum. Head similar to hexagon, HW 89% of HL. Corona slightly narrower than collar, with papillae clearly seen in the middle of each trochus, CW 97% of HW. Trochal discs separated by a narrow, V-shaped sulcus, in which a cylindrical ligula bends obliquely to the dorsal side (Fig. 4F, G). A slight contraction near the tip which then forms a small papilla on the tip of the ligula, attaining the level of the discs at the inner side (Fig. 4D, E). Upper lip a flat bow. Neck also bent ventrally when animal creeps. The first pseudosegment of neck slightly narrower than the head at the corners of the mouth, not distinct from head and trunk. A pair of lateral cuticular bulges on the dorsal antenna pseudosegment. Antenna with two segments, its length 30–40% of the bearing pseudosegment width.

Trunk slender and cylindrical, TrL 59–67% of TL. Rump conical, with both pseudosegments somewhat swollen and strong arched up dorsally and roofing the foot, the posterior rim of the second pseudosegment creased, RL 8–10% of TL.

Foot short with three pseudosegments, FL 6–8% of TL. Bulbous spurs short and triangular shape, with distinct tips and wide interspace, base swollen. The width of interspace 114% of SL, 97% of the swollen width. Three stout unsegmented toes of the same length. Trophi small, dental formula 3/3.

##### Measurements.

TL 186±43 μm, NL 27±3 μm, TrL 119±37 μm, RL 156±2 μm, RW 22±7, FL 12±2 μm, SL 4±2 μm, RaL 13±1 μm, TrW 5.6±0.5 μm.

##### Remarks.

*Habrotrocha ligula loxoglotta* was originally described from Holland (De Koning 1947), later reported from beech-oak needle-litter in Germany, from dry mosses in France (Donner 1951) and from mosses in Austria (Kutikova 2005). In this study, it was found for the first time in China (Yunnan Province) as well as in the Oriental region.

#### Habrotrocha serpens

Taxon classificationAnimaliaBdelloideaHabrotrochidae

Donner, 1949

54C1875B-173F-5A64-A69D-1089550BE4AD

Figure 5 ; Table 3

##### Materials.

Five specimens found in dry mosses on bark from southern China (GD6) (Table 2).

##### Description.

**Figure 5.**
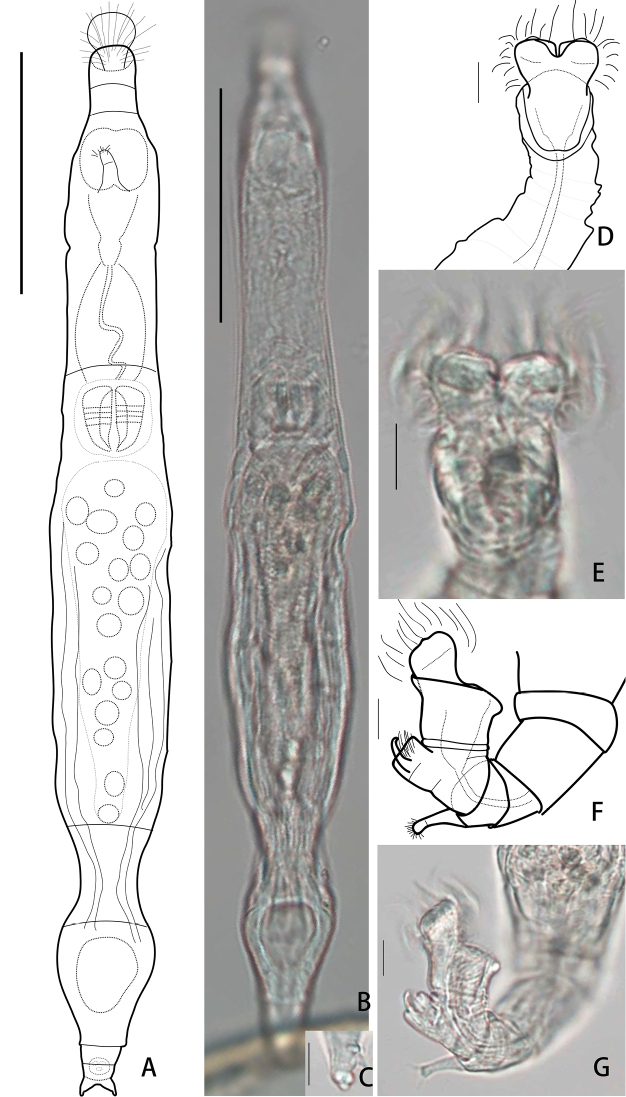
*Habrotrocha serpens* Donner, 1949 **A, B** habitus, creeping (not fully extended), dorsal view **C** three toes, dorsal view **D, E** head, ventral view **F, G** head, lateral view (the second pseudosegment of rostrum contracted). Scale bars: 50 μm (**A, B**); 10 μm (**C–G**).

Body extremely slender (BW is only about 6% of TL), long and cylindrical, integument transparent and smooth. Rostrum rather long, with two pseudosegments. The first pseudosegment circular and slightly bigger than the second one which often contracted (Fig. 5B). One whole semi-circular lamella not divided into lobes, rather large, broader than the rostrum, covers the long and stiff tactile cilia. Head slender, HW 44% of HL, 22% of TL. Corona also slender, a little wider than the head, CW 107% of HW. Trochal pedicels grown together, central rounded papillae on each separated trochal discs, incline to the dorsal side. Upper lip low, narrow and without lobes, slightly arched, not covered by the incompletely extended rostrum. Lower lip spoon-shaped, strongly protrudes forward.

Neck slender. Throat very short, pharyngeal tube long, undulating before the mastax. Dorsal antenna slender, with two segments, its length 86% of the antennal pseudosegment width. Trunk slender, the two lateral sides of trunk almost parallel when animal fully extended, the last trunk segment often strongly contracted. Rump conical, with both pseudosegments swollen, arched up dorsally and roofing the foot, RL 12% of TL.

Foot very short, of four pseudosegments, FL 5% of TL. Spurs triangular and have swollen base, each with curved inner margins and a very small interspace. SL 63% of SSW. Three short unsegmented and of approximately equal length toes (Fig. 5C). Trophi large, dental formula 4/4.

##### Measurements.

The detailed measurements are summarized in Table 5 with a comparison of the original data from Donner (1949; 1970).

**Table 5.** Comparison the body dimensions of *Habrotrocha serpens* between Chinese specimens and the original description from Donner (1949; 1970).

**Measurements**	**Chinese specimens**	**Donner 1949**	**Donner 1970**
TL	213	193–273	200
BW	18.7		17
HL	42		
HW	18.4		
CW	19.7		
NL	31.2		
MinNW	17.8		
MxNW	19.2		
RL	26.6		
RW	20		
FL	12		
FW	9.9		
SL	3.4		
SSW	5.4		
RaL	14	12.7	14.8
TrW	5.9		
TL/BW	11.4		11.8

BW: body width when creeping; FL: foot length; FW: foot width; HL: head length; HW: head width; CW: corona width; MinNW: minimal neck width; MxNW: maximal neck width; NL: neck length; RaL: ramus length; RL: rump length; RW: rump width; SL: spur length; SSW: spur pseudosegment width; TL: total length; TrW: trophi width. Measurements are given in μm.

##### Remarks.

The general morphology of our sample conforms with the description of the Austrian population except that the rostrum is not always fully expanded to/exceeding the upper lip in a feeding position. It may because of the second pseudosegment of rostrum often contracted. Additionally, we observed three approximately equal-lengthed toes which were not clear in Donner’s (1949) description.

This morphospecies was first described from soil from Austria by Donner (1949), and then recorded in moss and soil from Austria and Czechoslovakia (Bartoš 1951); in needle litter, *Calamagrosits* turf, grasses and leaf litter from Austria, Czechoslovakia, Romania, and Spain (Donner 1965, 1970). It is new for China as well as for the Oriental region.

## Discussion

### Taxonomy and diversity of bdelloid rotifers in China

Only 48 species were recorded in eleven studies conducted in China between 1908 and 2018 (Table 1), which implies that taxonomic and diversity researches on Chinese bdelloids are very limited. Moreover, only 65% (31 of 48) of the recoreded morphospecies were illustrated and described (e.g., Wang 1961; Bartoš 1963; Gong 1983), and many of the illustrations are inaccurate, not showing important details and the descriptions are not detailed enough to verify their identity. Besides, there are 17 morphospecies listed in the literature without any illustrations, photographs or descriptions (e.g., Koste and Zhuge 1996; Yin and Xu 2016), which need further verification. Also, some species were recorded out of their specific habitats (e.g., *H. thienemanni* Hauer and *P. roseola* Ehrenberg) and some recorded in unusual environments (e.g., *Habrotrocha pulchra* Murray, *H. thienemanni* Hauer, *Mniobia tentans* Donner, *Macrotrachela bullata* Murray, and *Philodina vorax* Janson were abundant in glacier over 5500 m a.s.l.) (Table 1). These ecological differences may hide potential cryptic taxa and need further studies combined with new techniques such as DNA taxonomy.

Due to a lack of insufficient taxonomic and diversity research in China, species richness is extremely uneven in different provinces of China. More morphospecies were recorded in the Tibetan Plateau (27 morphospecies) and Guangdong Province (22 morphospecies) with more samples collected (Stewart 1908; Bartoš 1963; Wang 1974; Gong 1983; Yin and Xu 2016). Four new morphospecies were reported in Guangdong, including *Habrotrocha modesta* Bartoš, *H. flexicollis* Bartoš, *Pleuretra proxima* Bartoš, and *P. similis* Bartoš. Unfortunately, they were never found again, and these are considered as disappeared ‘endemic morphospecies’ in latter researches. Research on different habitats of bdelloids were also uneven. Most studies were only focused on fragmented fresh-water bodies or mosses, but did not pay attention to other habitats such as brackish waters, soil and litter. Therefore, more studies are necessary to explore the taxonomy and diversity of bdelloid rotifers in China, especially with a focus on the areas and habitats that were not well studied.

### Geographical distribution and ecological information of Chinese bdelloids

The high dispersal potential of bdelloids has supposedly led to their generally cosmopolitan distribution (Fenchel and Finlay 2004). The previous extensive sampling of bdelloids confirms that some species can be found in distant areas on different continents, but also some species can only be found in specific area (Donner 1965; Segers 2007). At present, studies of biogeography on these taxa are not comprehensive. For example, *Adineta ricciae* Segers and Shiel, previously considered as an Australia-endemic species, was observed in South China (the Oriental region); *A. beysuanae* has been described in a container filled with plant debris and rain water from the United States (Örstan 2018), and it was then found in similar or drier habitats (dew and leaf litter) from China. These findings imply that the currently described distribution of bdelloids is incomplete and may be strong influenced by the sampling effort, especially in the poorly investigated areas, such as South Asia.

With study extending to more ecological habitats, some morphospecies were found in a broader range of habitats. We observed five brackish water morphospecies: *Rotaria rotatoria* Pallas, *R. laticeps* Wulfert, *R. tridens* Montet, *Philodina megalotrocha* Ehrenberg and *Macrotrachela hewitti* Murray. They were found among aquatic plants or brackish temporary puddle with sediment in mangrove. Noticeably, *R. rotatoria* was abundant and dominated in *Gracilaria lichenoides* (a red alga) culture ponds, possibly because *G. lichenoides* could provide suitable habitats. These ecological differences seem to represent different ecological niches, which may hide some interesting phenomena of separated evolutionary lineages. For example, *Adineta vaga*, which occurs in the multiple types of habitats, has a large amount of cryptic diversity (Fontaneto et al. 2011).

### More extensive surveys of bdelloids in Asia

More than half of the recorded morphospecies from this study (some presumed cosmopolitan) are new records for the Oriental region as well as for South Asia. As there are still considerable gaps in faunistic studies in the Oriental region, we do not yet have sufficient faunistic data to determine the true distributions of bdelloids. Our findings highlight the need for further taxonomic studies on bdelloids in Asia. Furthermore, asexual bdelloids have evolved independently in spite of being effectively sympatric, indicating that they may adapt to different ecological niches, thus the type of habitat is a key player for microscopic species diversity and evolution (Birky et al. 2005). Applications of molecular phylogeny for identification of bdelloid species would be invaluable in uncovering the actual systematic status of some euryoecious or variable morphospecies so that we may better understand the true distribution of bdelloid species.

## Supplementary Material

XML Treatment for Adineta cf. acuticornis

XML Treatment for Adineta beysunae

XML Treatment for Habrotrocha ligula loxoglotta

XML Treatment for Habrotrocha serpens
